# Sirtuin 3 deficiency aggravates contrast-induced acute kidney injury

**DOI:** 10.1186/s12967-018-1690-5

**Published:** 2018-11-16

**Authors:** Qinghai Zhang, Xun Liu, Na li, Jihong Zhang, Jianmin Yang, Peili Bu

**Affiliations:** 1 The Key Laboratory of Cardiovascular Remodeling and Function Research, Chinese Ministry of Education, Chinese National Health Commission and Chinese Academy of Medical Sciences, The State and Shandong Province Joint Key Laboratory of Translational Cardiovascular Medicine, Department of Cardiology, Qilu Hospital of Shandong University, Jinan, China; 2 Intensive Care Unit, Weifang People’s Hospital, Weifang, Shandong China

**Keywords:** Sirt3, Contrast-induced acute kidney injury, Reactive oxygen species, Apoptosis

## Abstract

**Background:**

Sirtuin 3 (Sirt3) is a key regulator of energy metabolism and oxidative stress. To investigate the role of Sirt3 in contrast-induced acute kidney injury (CIAKI), we established the model both in vivo and in vitro to explore the potential mechanisms.

**Methods:**

In vivo, we established CIAKI models in wild-type (WT) and Sirt3-knockout (Sirt3-KO) mice. Blood urea nitrogen (BUN) and serum creatinine (Scr) were detected by enzyme-linked immunosorbent assay, Glomerular Filtration Rate (GFR) and creatinine clearance were also investigated. We detected the production of reactive oxygen species (ROS) via 2′7′-dichlorodihydro-fluorescein diacetate. The expressions of Sirt3, oxidative stress and apoptosis related markers (MnSOD, Catalase, Acetyl-MnSOD K68, Nox4, Bax, Bcl-2 and Caspase3) were measured and analyzed. In addition, we observed the effect of nicotinamide riboside (NR) on CIAKI in WT and Sirt3-KO mice. In vitro, Sirt3 was knocked out by siRNA transfection method in HK-2 cells. Sirt3, ROS, oxidative stress and apoptosis markers in HK-2 cells were also measured.

**Results:**

Our data demonstrated that the levels of Scr and BUN in Sirt3-KO mice were increased while the levels of the GFR and creatinine clearance were decreased in CIAKI mice. In Sirt3-KO or siRNA groups, the activities of MnSOD and Catalase were markedly down-regulated. Also, the expression of Caspase3 were markedly increased and the ratio of Bcl-2/Bax was decreased, while the ROS level was increased in Sirt3 deficiency groups. NR ameliorated CIAKI in WT mice but not in Sirt3-KO mice.

**Conclusion:**

Our results suggest that Sirt3 deficiency aggravates contrast-induced acute kidney injury. Sirt3 is critical in NR-mediated renoprotection in CIAKI.

## Background

With the increasing use of contrast media in interventional procedures and diagnostic, contrast-induced acute kidney injury (CIAKI) has become the third leading cause of hospital-acquired acute renal failure, accounting for 10–25% of all acute renal failure cases [1], in spite of the introduction of safer and newer contrast media, the introduction of additional preventive strategies and the improvement of hydration protocols. Although a sufficient amount of fluid input may relieve contrast-induced AKI to some extent, accurate and effective therapeutic approaches is lacking. The pathogenesis of contrast media nephrotoxicity has not been better defined. The mechanisms underlying contrast media nephrotoxicity have not been fully elucidated and may be due to several factors, including renal ischemia, the formation of reactive oxygen species, reduction of nitric oxide production, and tubular epithelial and vascular endothelial injury [2]. In addition, contrast media can result in apoptosis and cell death of both endothelial and tubular cells due to the cytotoxicity caused by iodine [3].

Sirt3 is one of the seven mammalian sirtuins, localized in mitochondria, which area conserved family of proteins possessing NAD+-dependent deacetylase activity. Of the seven sirtuins, Sirt3 is the only sirtuin analogue whose increased expression was shown to be associated with longevity of humans [4]. Sirt3 has been found to play important roles in maintaining mitochondrial function and integrity in response to the oxidative stress. Sirt3 is a protein of tremendous potential, which can modulate a variety of cellular processes, including oxidative stress, ATP generation, metabolism, growth arrest, apoptosis and senescence by deacetylasing lysine residues of mitochondrial proteins. We have previously reported that Sirt3 attenuated angiotensin II-induced Myocardial remodeling [5, 6]. Recently, several results showed the protective role in kidney injury. Morigi et al. found that activation of Sirt3 attenuates mitochondrial dysfunction in cisplatin-induced acute kidney injury [7], and the results were confirmed by Liu et al. [8]. Another study found Sirt3 prevents renal tubulointerstitial fibrosis by ameliorating oxidative stress and mitochondrial dysfunction in an angiotensin II-induced kidney injury model [9]. Our newly study also found Sirt3 activation ameliorates kidney injury induced by hypertension [10]. Despite these research efforts, whether endogenous Sirt3 play a protective role in CIAKI is still unclear. The current study was designed to test the hypothesis that endogenous Sirt3 may play a protective role in CIAKI and Sirt3 deficiency may aggravate CIAKI in mice. In the present study, we performed a series experiments to determine whether Sirt3 deletion enhances CIAKI and reveals the evidence that endogenous Sirt3 prevents CIAKI in mice. The oxidative stress and apoptosis indices were also investigated as the potential mechanisms for the protection. Taken together, the results show for the first time whether endogenous Sirt3 produces protection against CIAKI in mice.

In the current study, we performed a series of experiments in vitro and in vivo to study the role and the essential mechanism of Sirt3 in CIAKI. Taking together, our findings suggest that Sirt3 deletion may exacerbate contrast-induced acute kidney injury and renal tubular epithelial cells apoptosis in a mouse model of CIAKI as well as in vitro.

## Materials and methods

### Animal model

Male global Sirt3-KO (129-Sirt3tm1.1Fwa/J) mice about 8 weeks old were purchased from Jackson Laboratory (USA). 129 wild-type (WT) mice were obtained from Department of Laboratory Animal Science of Peking University (Beijing, China). All animals were housed in standard cages and kept on a 12-h light/12-h dark cycle with food and water freely available.

In the first part of the in vivo study, the experimental mice were randomly divided into 4 groups (n = 8 in each group): WT + vehicle group, Sirt3-KO + vehicle group, WT + Ioversol group, Sirt3-KO + Ioversol group. CIAKI was induced in mice as described previously [11]. In brief, after 16 h water deprivation and prior inhibition of prostaglandin and nitric oxidative synthesis, mice in Ioversol-treated groups were injected subcutaneously with the Ioversol (3 mg/g organically bound iodine, Hengrui medicine, Ltd, Jiangsu, China). To inhibit the cyclooxygenase and nitric oxide synthase, mice were injected with indomethacin (10 µg/g; Sigma-Aldrich, US), and *N*^G^-nitro-l-arginine methyl ester (l-NAME, 10 µg/g; Sigma-Aldrich, US). Mice in control groups received subcutaneous injections of saline, instead of Ioversol, after indomethacin and l-NAME injection and 16 h of water deprivation. The animals were euthanized 24 h later using an overdose of pentobarbital sodium for biochemical and histopathological examinations.

In the second part of the in vivo study, in order to assess the effect of Sirt3 activator nicotinamide riboside (NR) on CIAKI, mice were divided into 4 groups (n = 8 in each group): WT + Ioversol group, Sirt3-KO + Ioversol group, WT + Ioversol + NR group, Sirt3-KO + Ioversol + NR group. Mice were injected twice daily with NR (1000 mg/kg) for 5 days until they were sacrificed as described previously [12].

### Renal function

The levels of serum BUN and Scr were measured by ELISA kits based on the manufacturer’s operation manual. The glomerular filtration rate (GFR) was estimated using fluorescein isothiocyanate (FITC-inulin, Sigma) technique as previously described [13].

### Kidney immunohistochemical staining

The renal tissues were embedded in paraffin and Sections (5 μm) were stained with hematoxylin and eosin (HE) to evaluate renal morphology. Histopathological analysis was performed by an experienced pathologist in our institute, who was blinded to the groups when the slides were evaluated. The renal morphology characteristics including the interstitial edema, cytoplasmic vacuolar changes and intratubular cast formation were estimated according to Billings et al. [14]. The primary antibody against Bax, Bcl-2, Caspase3 and NOX4 were used for immunohistochemical staining. Dihydroethidium (DHE) (2 μM) was used as previously described to test superoxide in frozen sections [15]. DCFH-DA (Beyotime, Jiangsu, China) test kit was used for the assessment of ROS. In brief, HK-2 cells were stained with 10 µmol/l DCFH-DA and then cultured in an incubator (37 °C, 5% CO_2_) for 20 min in dark. After washing three times with PBS, cells were exposed to 100 mg/ml-iodine Ioversol for 30 min. A confocal laser scanning microscope (ZEISS LSM710, Germany) was used to detect cells with excitation at 488 nm and an emission at 520 nm.

### SOD2 and catalase activities

SOD2 activity was determined by a Cu/Zn-SOD and Mn-SOD Assay Kit with WST-8 (Beyotime, China) according to the manufacturer’s instructions [16]. One unit of SOD2 activity was defined as the amount of SOD2 needed to exhibit 50% dismutation of the produced superoxide radical. The final enzyme activity was calculated by normalizing the results to the total protein concentration of the whole protein extract. Catalase (CAT) was determined by the method and the activity of CAT is expressed as µmoles of H_2_O_2_ decomposed/min/mg protein or U/mg protein [17].

### Cell culture

HK-2 cells were cultured in 6-wells plates (Corning, NY) and maintained in low glucose Dulbecco’s Modified Eagle’s medium (Gibco) containing 10% fetal bovine serum (FBS and 1:100 penicillin/streptomycin in a humidified incubator (37 °C, 5% CO_2_). Cells were subcultured twice a week by harvesting with trypsin/EDTA and seeding in 75 cm^2^ flasks. Cells from passages 4–8 at 80% confluence in culture wells were used after 24 h serum depletion prior to treatment. Cells were divided into 4 groups: HK-2 + vehicle group, HK-2 Sirt3-siRNA + vehicle group, HK-2 + Ioversol group and HK-2 Sirt3-siRNA + Ioversol group. For Sirt3 silencing in vitro, lipofectamine 2000 (Invitrogen, Carlsbad, USA) was employed to transfect the HK-2 cells with 50 nM of small interfering RNA (siRNA) following the manufacturer’s instructions. The sequence of siRNA Sirt3 is (5′-3′) GCGUUGUGAAACCUGACAUTTAUGUCAGGUUUCACAACGCTT. The Sirt3 siRNA and negative control siRNA was prepared by Genepharma (Shanghai, China). In Ioversol-treated groups, cells transfected with Sirt3 siRNA for 24 h and then were exposed to 320 mgI/ml Ioversol for 30 min, followed by incubation for 24 h without Ioversol.

### Western blot analysis

In order to detect the protein levels of Sirt3, Acetyl-MnSOD K68, Nox4, Bax, Bcl-2 and Caspase3, total proteins of the kidneys of the mice or the cultured HK-2 cells were isolated and purified with cell lysis buffer (100 mM Tris–Cl, pH 6.8, 4%(m/v) SDS, 20% (v/v) glycerol, 200 mM β-mercaptoethanol, 1 mM PMSF, and 1 g/ml Aprotinin). Solubilized protein was separated by electrophoresis and transferred to nitrocellulose membranes. Membranes were probed with the specific antibodies followed by incubation with horseradish peroxidase-conjugated secondary antibodies. GAPDH immunoblot analysis was performed to ensure equal sample loading. The immunoreactive bands were visualized using enhanced chemiluminescence detection system (Pierce) and quantified by densitometry in accordance with the manufacturer’s instructions.

### Cell apoptosis

Cell apoptosis was assessed by TUNEL assay. The kidney tissues were cut into serial 5 µm sections and stained following the manufacturer’s instructions. The rate of apoptosis was evaluated by counting numbers of TUNEL-positive cells under ×400 magnifications.

### Statistical analysis

Statistical analyses were analyzed using Graphpad Prism (version 5.00 for windows, GraphPad Software). The student’s two-tailed t-test was used to evaluate the between-group comparisons difference. A *p* value of less than 0.05 was considered statistically significant difference. All of the experimental data were expressed as mean ± SEM.

## Results

### Sirt3 protein expression increased in contrast-induced acute kidney injury in vivo and in vitro

**Fig. 1:**
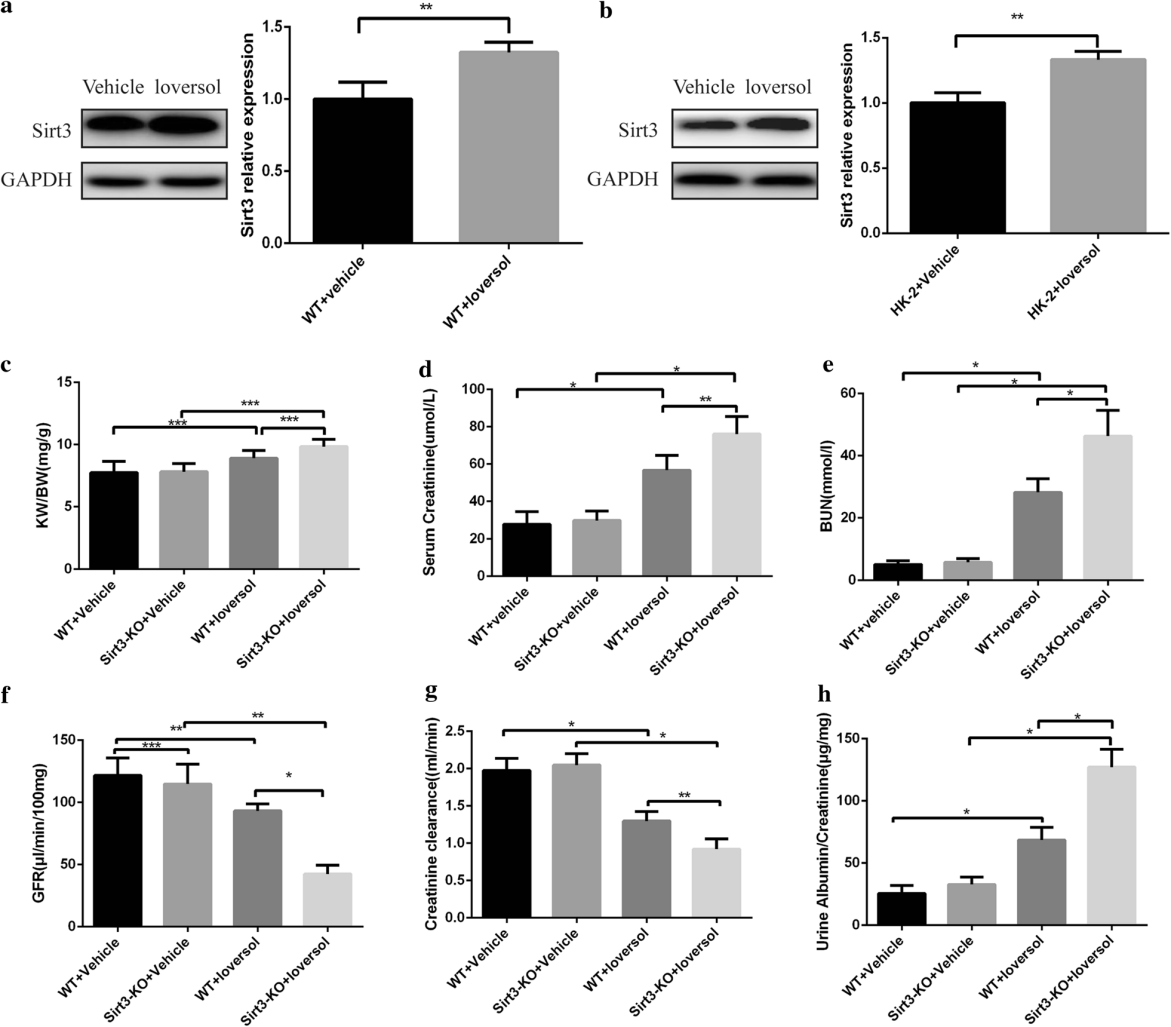
Sirt3 deficiency worsened renal function in contrast-induced acute kidney injury. **a** Representative Western blot and quantitative analysis in mice. **b** Representative Western blot and quantitative analysis in HK-2 cells. **c** Ratio of the kidney weight to body weight in different groups. **d**–**h** The levels of serum creatinine, blood urea nitrogen, glomerular filtration rate, creatinine clearance and ratio of urine albumin to creatinine in different groups. **p < 0.05,*p < 0.01,***p > 0.05

First, we tested the sirt3 expression in contrast-induced acute kidney injury model in vivo and in vitro. We found that Ioversol treatment significantly increase the Sirt3 expression in WT mice (Fig. 1a) and HK-2 cells (Fig. 1b), suggesting the potential role of Sirt3 in CIAKI.

### Sirt3 deficiency aggravated renal function in CIAKI model

To investigate the effect of Sirt3 deficiency on acute kidney injury, we first weighed the body and kidney of mice in all groups and found that the ratio of kidney weight to body weight (KW/BW) was increased in Ioversol groups, however, the difference did not reach statistically significance (Fig. 1c). However, the levels of Scr and BUN were remarkably elevated in the CIAKI mice after 24 h, compared with the controls. Two model groups both developed acute kidney injury, while Sirt3-KO mice demonstrated more serious renal dysfunction. Ioversol treatment increased the levels of Scr, BUN and the ratio of urine albumin to creatinine, and Sirt3 deficiency further increased the levels of the above index (Fig. 1d, e, h). Accordingly, the GFR and creatinine clearance, were decreased in mice after Ioversol infusion, while the tendency of the ratio of albumin to urine creatinine was opposite to those of GFR and creatinine clearance (Fig. 1f, g). Consider together, the changes of Scr, BUN, GFR, creatinine clearance and the ratio of urine albumin to creatinine showed that Sirt3-KO mice developed more serious kidney injury, indicating that Sirt3 may be involved in CIAKI.

### Sirt3 deficiency worsen the renal histologic injury

**Fig. 2:**
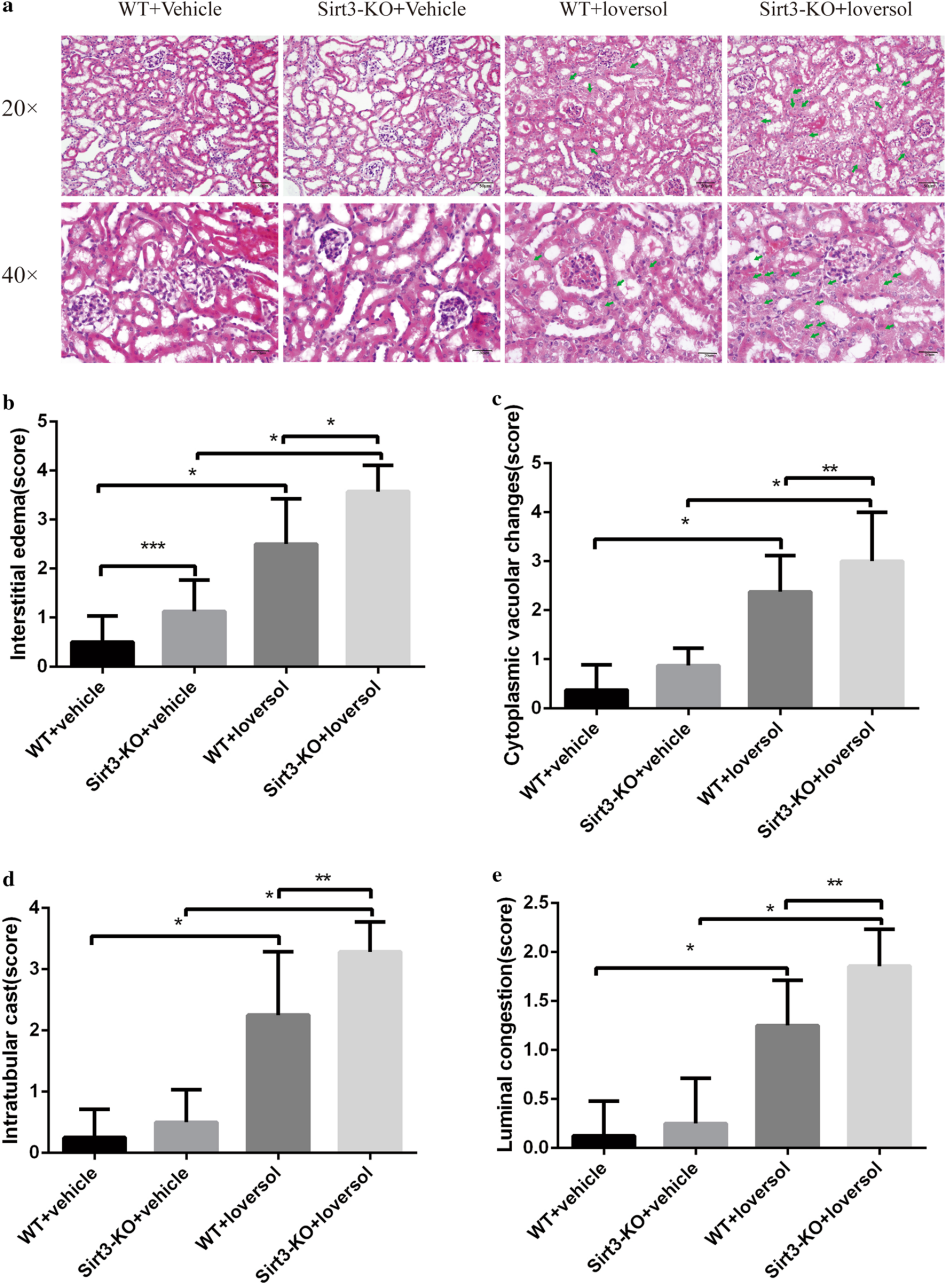
Histopathological HE staining in kidney tissues. **a** Representative photomicrographs of HE-stained kidney sections are presented as indicated by the green arrows. **b**–**e** Semi-quantitative analysis of interstitial edema, cytoplasmic vacuolar changes, intratubular cast formation and luminal congestion of the picture ×400. **p < 0.05, *p < 0.01, ***p > 0.05

The Ioversol groups exhibited markedly changes in kidneys with HE staining compared with the vehicle groups (Fig. 2). Architectural injuries, including luminal congestion, cytoplasmic vacuolar changes, intratubular cast formation, and the interstitial edema in the renal tubular were observed in the Ioversol-treated WT mice. However, Sirt3-KO mice with the Ioversol treatment developed aggravated renal tubular injury. These results indicated that endogenous Sirt3 might have a protective effect against acute kidney injury induced by contrast medium.

### Sirt3 deficiency exacerbated oxidative stress in CIAKI model

**Fig. 3:**
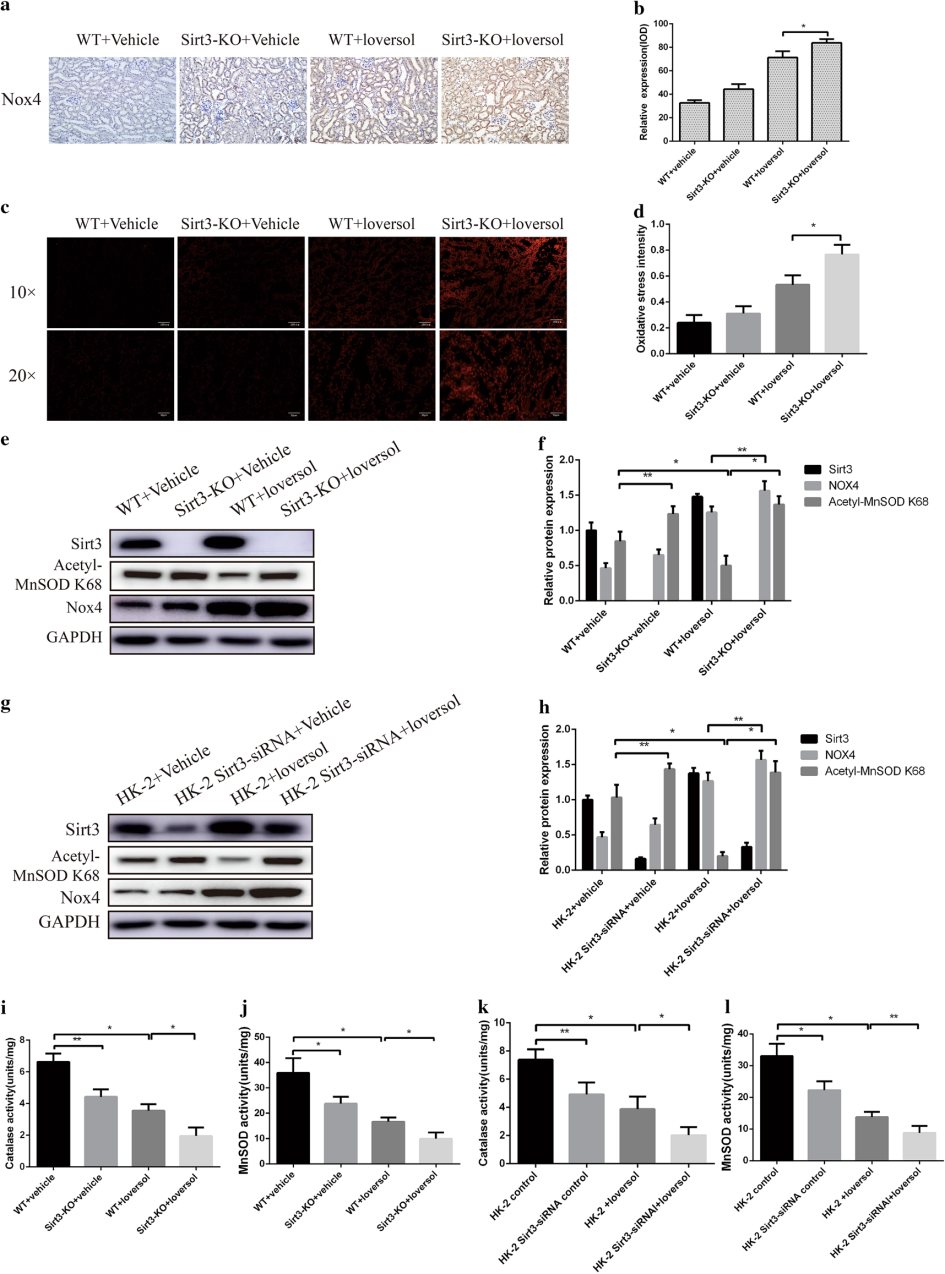
Sirt3 deficiency aggravated oxidative stress in contrast-induced acute kidney injury. **a** and **b** Immunohistochemical staining and quantitative analysis of Nox4 in murine kidney. **c** and **d** Representative dihydroethidium staining and quantitative analysis in murine kidney. **e** and **f** Representative Western blot and quantitative analysis of Sirt3, Nox4 and acetyl-MnSOD K68 in murine kidney. **g** and **h** Representative Western blot and quantitative analysis of Sirt3, Nox4 and Acetyl-MnSOD K68 in HK-2 cells. **i** and **j** Catalase and MnSOD activities in murine kidney. **k** and **l** Catalase and MnSOD activities in HK-2 cells. **p < 0.05, *p < 0.01

Oxidative stress is one of the essential procedures of contrast medium induced acute kidney injury. Nox4 plays an important role in oxidative stress and inflammatory reaction, so we tested its expression and found that Ioversol increased Nox4 levels and Sirt3 deficiency exacerbated this tendency (Fig. 3a, b, e, f). Similarly, superoxide levels were also elevated in Ioversol-treated groups and further raised in Sirt3 deficiency groups (Fig. 3c, d). Acetyl-MnSOD K68 is often used as a marker for Sirt3 activity. Our results found that Sirt3 deletion increased the level of Acetyl-MnSOD K68, and loversol decrease Acetyl-MnSOD K68 expression in WT mice while had no effect on Acetyl-MnSOD K68 expression in Sirt3 deficiency mice (Fig. 3e, f). The changes of Nox4 and Acetyl-MnSOD K68 in vitro were similar with the in vivo results (Fig. 3g, h). In addition, Ioversol decreased Catalase and MnSOD activities and Sirt3 deficiency exacerbated these trends (Fig. 3i, j, k, l). Altogether, Sirt3 deficiency may aggravate oxidative stress in contrast-induced acute kidney injury.

### Sirt3 deficiency elevated Ioversol-induced ROS generation in HK-2 cells

**Fig. 4:**
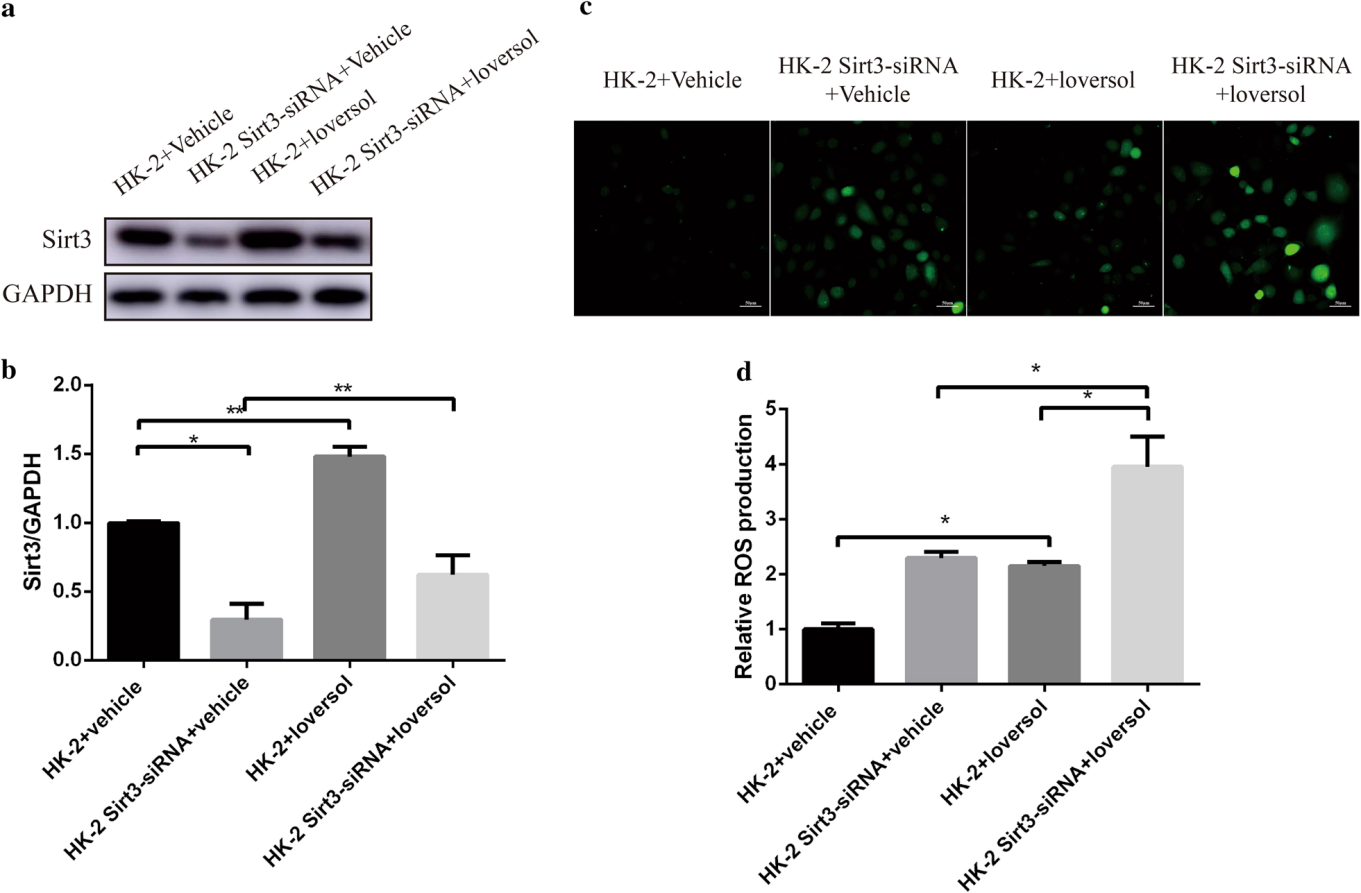
The effect of Sirt3 deficiency on ROS level in HK-2 cells treated with Ioversol. **a** Representative Western blot staining of Sirt3. **b** Quantitative analysis of **a**. **c** ROS staining in HK-2 cell. **d** Quantitative analysis of C. **p < 0.05,*p < 0.01

Firstly, we confirmed that the expression of Sirt3 was decreased in HK-2 cell pretreated with Sirt3 siRNA compared with the controls (Fig. 4a, b). Our results demonstrated that loversol significantly increased the ROS level and Sirt3 deficiency also enhanced ROS expression. These results showed that Sirt3 deficiency increase the ROS level of HK-2 cell induced by contrast medium.

### Sirt3 exerted a protective effect against apoptosis in CIAKI model

**Fig. 5:**
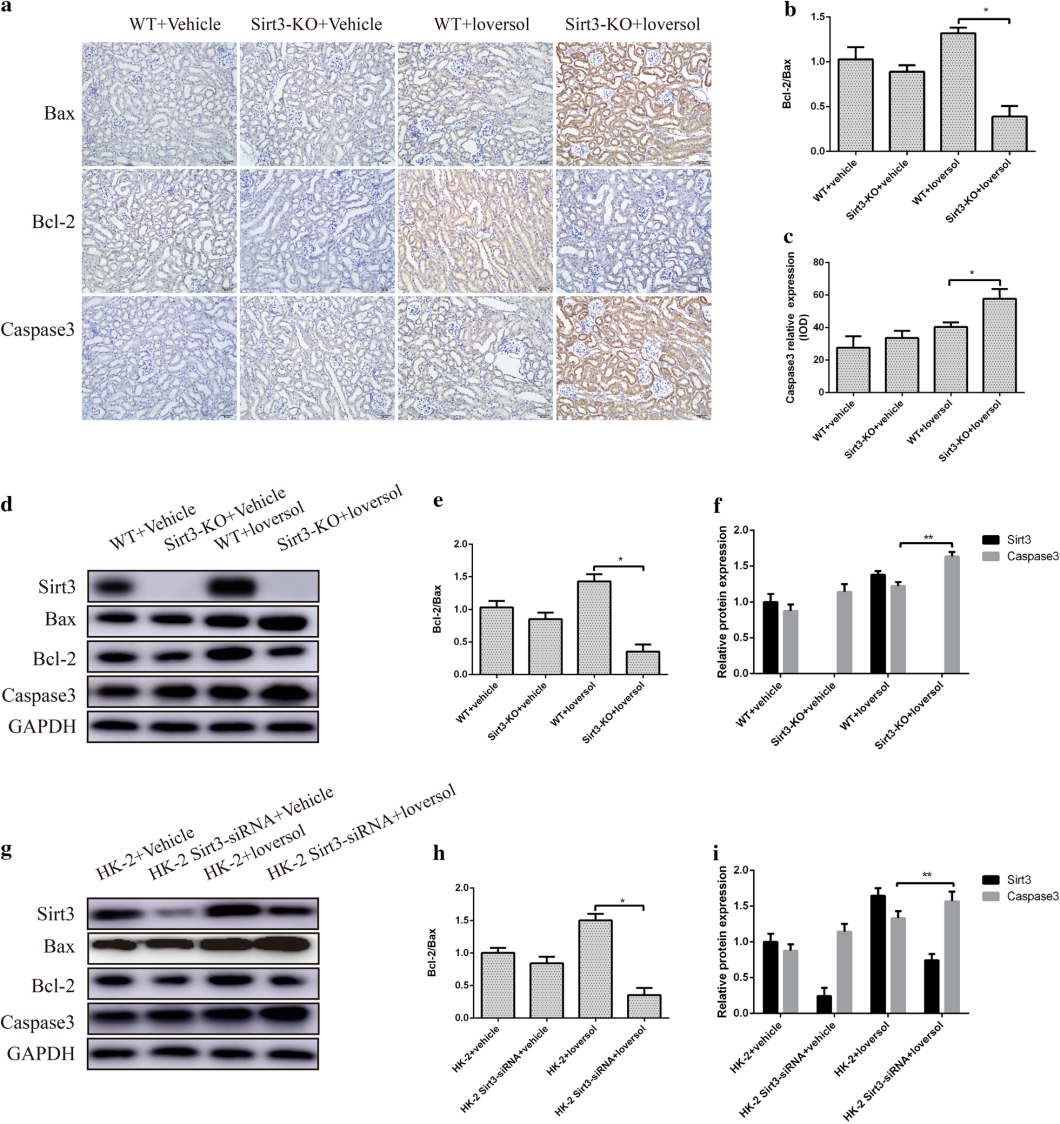
Sirt3 deficiency increased apoptosis in contrast-induced acute kidney injury. **a** Immunohistochemical analysis of Bax, Bcl-2 and Caspase3 in murine kidney. **b** and **c** Quantitative analysis of Caspase3 and Bcl-2/Bax in murine kidney. **d** Representative Western blot analysis of Bax, Bcl-2 and Caspase3 in murine kidney. **e** and **f** Quantitative analysis of Caspase3 and Bcl-2/Bax in murine kidney. **g** Representative Western blot analysis of Bax, Bcl-2 and Caspase3 in HK-2 cell. **h** and **i** Quantitative analysis of Caspase3 and Bcl-2/Bax in HK-2 cells. **p < 0.05, *p < 0.01

Apoptosis in the kidney is linked to the severity of AKI. To further determine the role of Sirt3 in CIAKI, we evaluated the expression of apoptotic proteins and anti-apoptotic proteins in our model. We firstly evaluated the Bax, Bcl-2 and Caspase3 expressions in kidneys using immunohistochemistry assay. Compared with the WT mice, the expression Caspase3 was markedly increased in Sirt3-KO mice after Ioversol treatment, and the changes affected by Sirt3 were of notably statistical significance. Besides, Sirt3 deficiency significantly decreased the ratio of Bcl-2 to Bax in Ioversol-treated mice (Fig. 5a–c). Western blot also revealed the similar results (Fig. 5d–f). In addition, we confirmed the above results in HK-2 cells (Fig. 5g–i). Taken together, Sirt3 deficiency aggravated apoptosis in kidney of CIAKI model.

### Sirt3 deficiency enhanced apoptosis in contrast-induced acute kidney injury

**Fig. 6:**
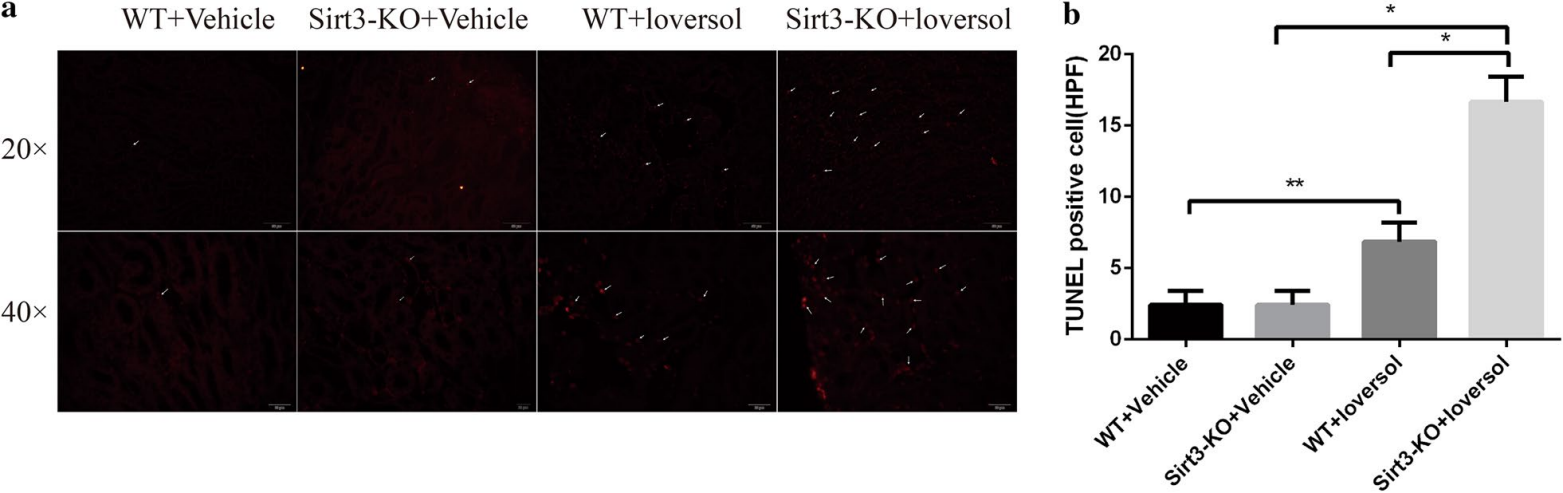
The effect of Sirt3 on apoptosis in kidney treated with Ioversol. **a** Tunnel staining in murine kidney. **b** Quantitative analysis of **a**. *p < 0.01

Cell apoptosis was evaluated by terminal deoxynucleotide transferase-mediated uridine triphosphate (dUTP) nick-end labeling (TUNEL) immunostaining. Our current results showed that TUNEL-positive tubular cell numbers were markedly increased in the kidneys of Sirt3-KO mice after treating with Ioversol (Fig. 6a, b). These results indicated that Sirt3 deficiency increased apoptosis in kidney of Ioversol-treated mice.

### NR attenuated contrast-induced acute kidney injury in WT mice but not in Sirt3 deficiency mice

**Fig. 7:**
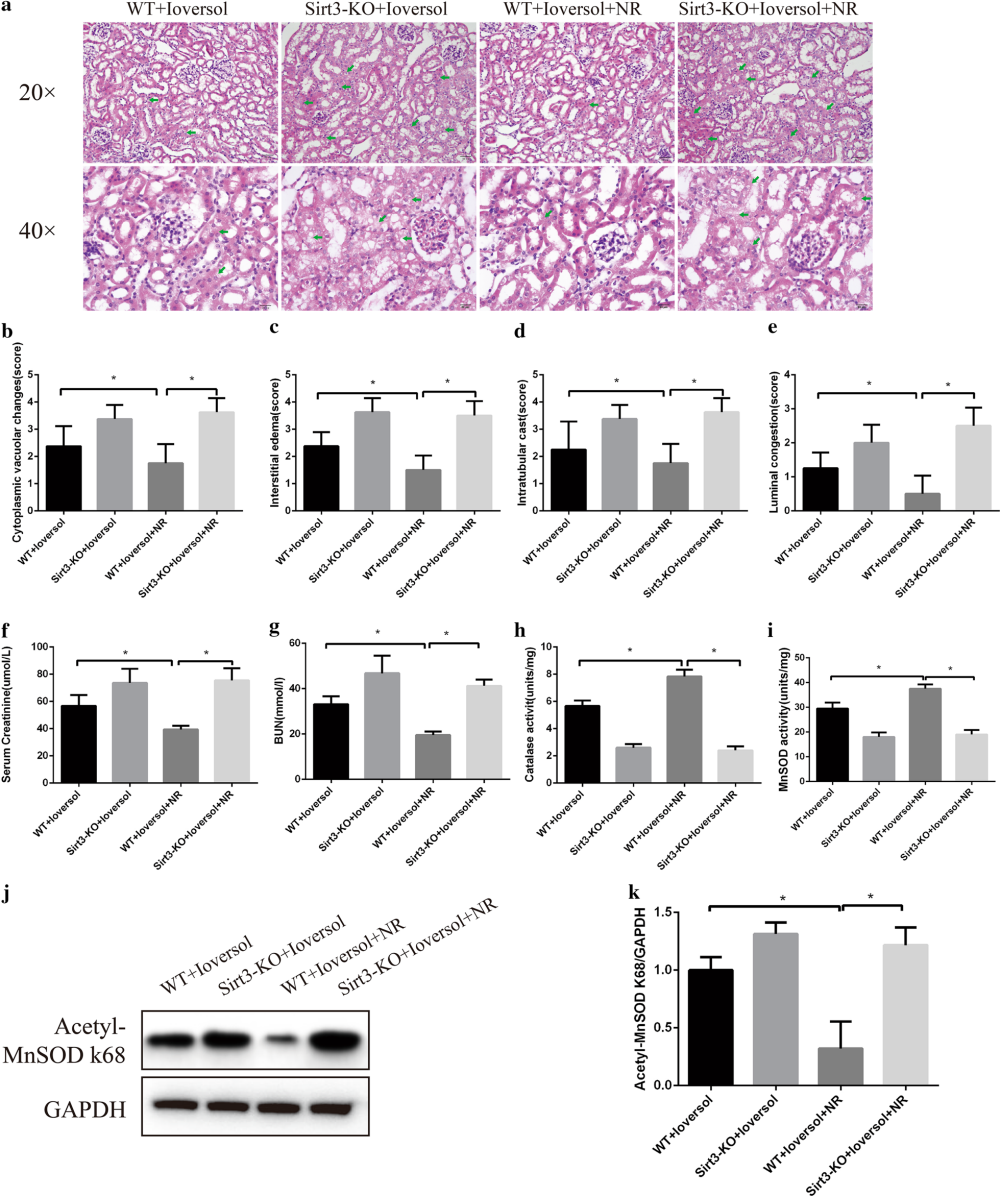
The effect of Nicotinamide riboside (NR) in contrast-induced acute kidney injury. **a** Representative HE-stained kidney sections are presented as indicated by the green arrows. **b**–**e** Semi-quantitative analysis of interstitial edema, cytoplasmic vacuolar changes, intratubular cast formation and luminal congestion of the picture ×400. **f** and **g** Serum creatinine and blood urea nitrogen levels. **h** and **i** Catalase and MnSOD activities in murine kidney. **j** and **k** Representative Western blot analysis and quantification of acetyl-MnSOD K68 in murine kidney.**p < 0.05

Our results demonstrated that the architectural injuries, including cytoplasmic vacuolar changes, interstitial edema, severe intratubular cast formation, and luminal congestion in the renal tubular were observed in the Ioversol-treated WT mice, and NR ameliorated these above injuries in WT mice but not in Sirt3 deficiency mice. Similarly, NR treatment decreased the levels of Scr and BUN, and increased the activities of catalase and MnSOD in WT mice, while did not alter these above index in Sirt3 deficiency mice. In addition, NR decreased Acetyl-MnSOD K68 expression in WT mice while had no effect on acetyl-MnSOD K68 expression in Sirt3 deficiency mice (Fig. 7).

## Discussion

Although the mechanisms underlying CIAKI have not been adequately clarified, studies have shown that overactivated oxidative stress is one of the key pathological manifestations. Oxidative stress, defined as disturbances in the pro-/antioxidant balance, acted as an important role in the procedure of CIAKI [18]. Boyacioglu et al. [19] found that oxidative stress related enzyme, such as MnSOD and catalase, were significantly improved with l-carnitine therapy. A research has reported that Sirt3 could prevent cardiac hypertrophic response by eliminating ROS level via up-regulating MnSOD and CAT [20]. In our study, the significantly decreased activities of MnSOD and Catalase were shown in Sirt3-KO mice compared with the wild-type mice after Ioversol treatment in kidneys. It has been showed that Nox4 is the classic pathway mediating oxidative stress and inflammation. In our study, the level of Nox4 was significantly decreased in the kidney tissues in Sirt3 deficiency mice relative to the WT mice after Ioversol treatment. Moreover, the results were consistent in HK-2 cells. These results in molecular biological parameters may explain that more serious oxidative stress occur in Sirt3-KO mice. The above results showed that endogenous Sirt3 exerted antioxidative effects to protect renal tubular cells against contrast-induced acute kidney injury.

ROS, mainly produced in the mitochondria, participates in the regulation of mitochondrial metabolism during oxidative phosphorylation [21]. The elevation in oxidative stress is observed with contrast medium and is probably related to the presence of iodine, which is a known inducer of ROS. Moreover, Sirt3 is emerging as a pivotal regulator of oxidative stress by deacetylation of substrates involved in ROS production [22]. Most importantly, it has been reported that Sirt3 is an essential key to lower cellular ROS level under the oxidative stress [23]. Our present study demonstrated that Sirt3 siRNA significantly increased ROS production in HK-2 cells, indicating that Sirt3 deficiency disturbed the pro-/antioxidant balance and boosted oxidative stress in CIAKI.

Experimental and clinical studies have shown that apoptosis is also considered to be a key regulator of acute kidney injury. Apoptosis is occurred in various procedures such as ischemia and anoxia, inflammatory reactions and drug toxicity damage [19]. Apoptotic genes and the caspase cycle are considered to be responsible for apoptosis. Recent reports have demonstrated that the level of caspase-3, Bax and Bcl-2 play important roles in the apoptosis procedure of initiation and maintenance [24]. In our study, we found that Sirt3 deficiency significantly decreased the ratio of Bcl-2 to Bax and increased caspase-3 expression in Ioversol-treated mice. Similar results were also found in HK-2 cells. In addition, TUNEL staining also demonstrated that Sirt3 deficiency aggravated kidney tissues apoptosis in mice treated with Ioversol. The above results showed that Sirt3 deficiency accelerated apoptosis in contrast-induced acute kidney injury.

Previous studies reported that activation of Sirt3 by the NAD^+^ precursor nicotinamide riboside (NR) protects from noise-induced hearing loss [12]. However, little is known whether NR could ameliorate CIAKI by activating Sirt3. Our current study found that NR treatment attenuated renal tubular injury, increased catalase and MnSOD activities, and ameliorated kidney function in WT mice but not in Sirt3 deficiency mice. In addition, Acetyl-MnSOD K68 is often used for Sirt3 activity assessment, and we found that NR decreased acetyl-MnSOD K68 expression in WT mice while had no effect on acetyl-MnSOD K68 expression in Sirt3 deficiency mice. These results suggested that Sirt3 might play a vital role in NR-mediated protective effect on CIAKI.

## Conclusion

In conclusion, our study indicates that Sirt3 deficiency aggravated renal injury in CIAKI. Sirt3 is critical in NR-mediated renoprotective effect in CIAKI. Thus, Sirt3 might provide a new therapeutic target to protect kidney injury against contrast medium.

## Abbreviations

Sirt3: Sirtuin 3
CIAKI: contrast-induced acute kidney injury
WT: wild-type
Sirt3-KO: Sirt3-knockout
Scr: serum creatinine
ELISA: enzyme-linked immunosorbent assay
GFR: glomerular filtration rate
ROS: reactive oxygen species
DCFH-DA: 2′7′-dichlorodihydro-fluorescein diacetate
