# Correction to: Sirtuin 3 deficiency aggravates contrast-induced acute kidney injury

**DOI:** 10.1186/s12967-021-03172-y

**Published:** 2022-01-28

**Authors:** Qinghai Zhang, Xun Liu, Na li, Jihong Zhang, Jianmin Yang, Peili Bu

**Affiliations:** 1 The Key Laboratory of Cardiovascular Remodeling and Function Research, Chinese Ministry of Education, Chinese National Health Commission and Chinese Academy of Medical Sciences, The State and Shandong Province Joint Key Laboratory of Translational Cardiovascular Medicine, Department of Cardiology, Qilu Hospital of Shandong University, Jinan, China; 2 Intensive Care Unit, Weifang People’s Hospital, Weifang, Shandong China

## Correction to: J Transl Med (2018) 16:313 10.1186/s12967-018-1690-5

The authors of the original article [1] have found out after publication that an incorrect panel was used in Fig. 2a. This affected the image of Sirt3-KO + loversol (40 ×).

These changes don’t affect any conclusions of the original paper. The Correct and incorrect figures are shown in this correction article (Figs. 1 and 2). The original article has been updated.

**Fig. 1:**
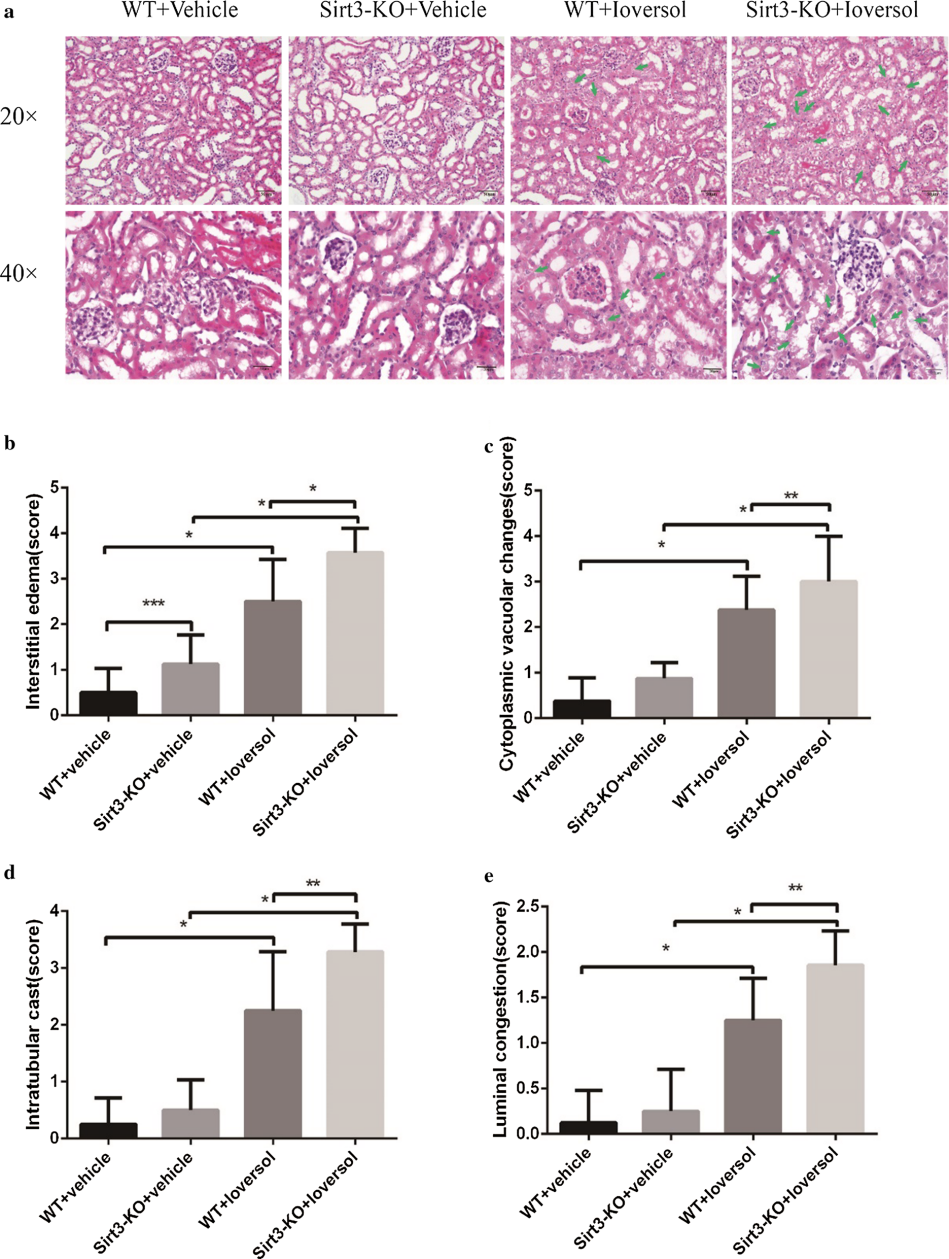
Correct version of Fig. 2a

**Fig. 2:**
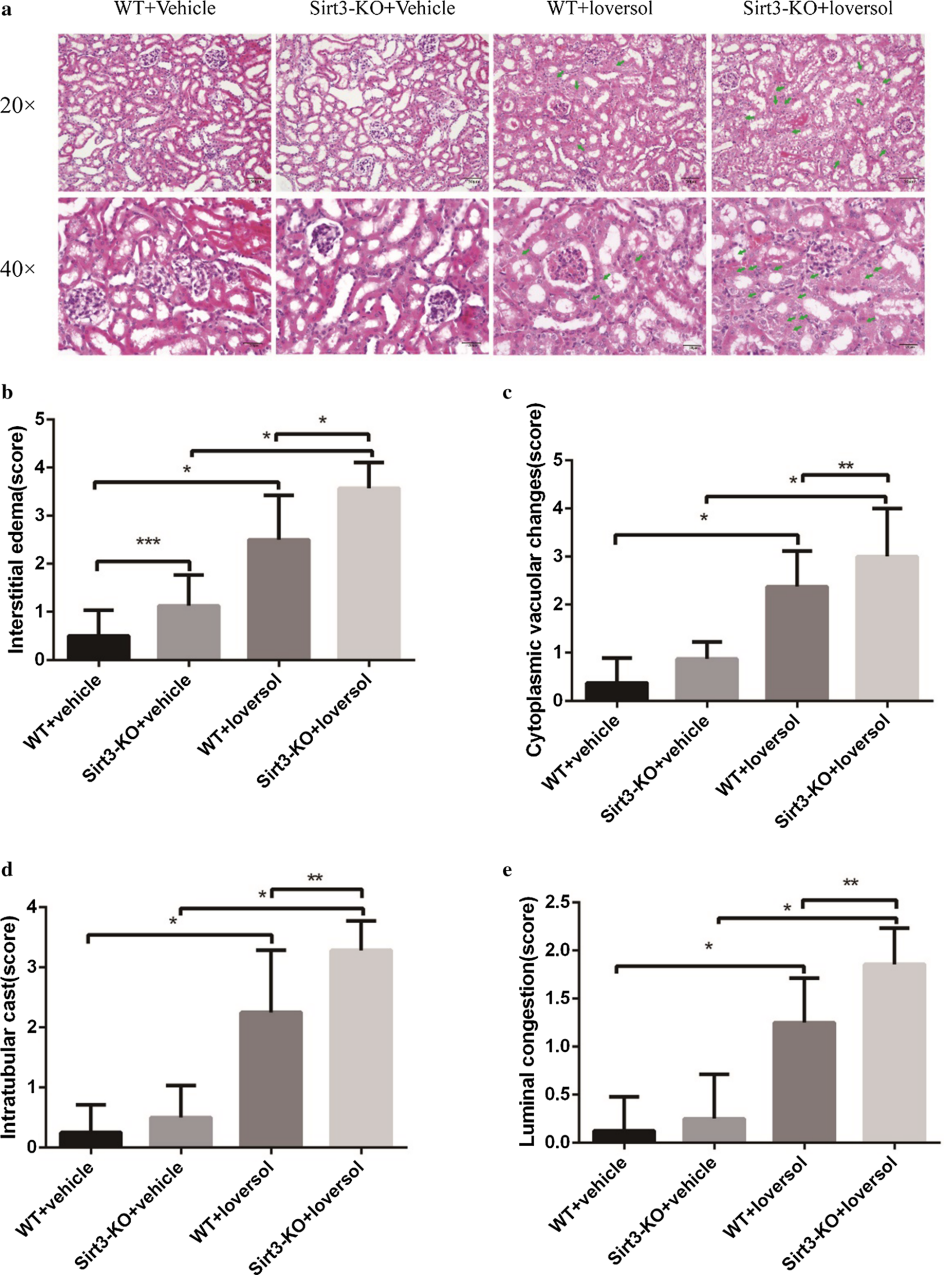
Incorrect version of Fig. 2a
